# Evolutionary conservation of leptin effects on wound healing in vertebrates: Implications for veterinary medicine

**DOI:** 10.3389/fendo.2022.938296

**Published:** 2022-08-25

**Authors:** Robyn E. Reeve, Kyla Quale, Grace H. Curtis, Erica J. Crespi

**Affiliations:** School of Biological Sciences and Center for Reproductive Biology, Washington State University, Pullman, WA, United States

**Keywords:** *Xenopus*, leptin, wound healing, JAK/STAT, injury, skin, nerve, amphibians

## Abstract

In mammals, the cytokine hormone leptin promotes wound healing by increasing inflammation, cellular recruitment, angiogenic regrowth, and re-epithelialization; however, it is not known whether leptin has conserved actions on wound healing in other vertebrates. Here, we tested the hypothesis that leptin promotes both the quality and speed of wound healing in the South African clawed frog, *Xenopus laevis*. First, fluorescent immunohistochemistry using a polyclonal antibody specific to *Xenopus* leptin showed that in juvenile dorsal skin, leptin protein is expressed in the dorsal epidermal layer, as well in blood vessel endothelial cells and sensory nerves that run along the base of the dermis. Injection of recombinant *Xenopus* leptin (rXleptin) stimulates phosphorylated STAT3 (pSTAT3), indicative of leptin-activated JAK/STAT signaling in the epidermis. Similar to mammals, leptin protein expression increases at the wound site after injury of the epidermis. We then cultured “punch-in-a-punch” full-thickness dorsal skin explants in three doses of rXleptin (0, 10, and 100 ng/ml) and showed that leptin treatment doubled the rate of wound closure after 48 h relative to skin punches cultured without leptin. Food restriction prior to wound explant culture reduced the amount of wound closure, but leptin injection prior to euthanasia rescued closure to similar control levels. Leptin treatment also significantly reduced bacterial infection of these epidermal punches by 48 h in culture. This study shows that leptin is likely an endogenous promoter of wound healing in amphibians. Leptin-based therapies have the potential to expedite healing and reduce the incidence of secondary infections without toxicity issues, the threat of antibiotic resistance, or environmental antibiotic contamination. The conservation of leptin’s actions on wound healing also suggests that it may have similar veterinary applications for other exotic species.

## Introduction

As the number of important amphibian populations grows in breeding and conservation collections in zoos, conservation programs, and the pet industry, there is a recognized need for better therapies for the treatment of injuries and infections (1–3). In amphibians, the skin is an important organ for respiration and immunity, but it is also easily injured and vulnerable to infection. Current treatments for skin wounds or diseases in captive amphibians are limited to antibiotics and antifungals, though pharmacokinetics and effective dosages are not well understood (4–6). While these are effective at preventing infection, they do not explicitly accelerate wound healing. Overuse of antibiotics also contributes to increased antibiotic resistance, and the persistence of these compounds in water systems is also of concern (7). Thus, there is a need for alternative treatments to help prevent infection and increase wound healing in these animals.

Understanding wound healing in amphibians also has biomedical applications. In all vertebrates, epidermal wound healing is a complex process that requires coordination of local and systematic immune responses, followed by tissue regrowth and remodeling events such as re-epithelization and re-vascularization (8). However, in most species, this process ends with a scar due to imperfect reorganization of the healed tissue. *Xenopus* frogs are capable of scarless wound healing as larvae and froglets but lose this ability over time, and, by 15 months post-metamorphosis, wound healing occurs with more fibrotic scar tissue (9). This change may be because of a stronger and longer-lasting inflammatory response in adult frogs, as the immune system takes up to a year to mature after metamorphosis (10), or because of skin maturation, as changes in skin makeup are associated with increased scarring in adult mammals (11). Thus, understanding the alteration in wound healing capacity as maturation occurs in amphibians could lead to therapies for scarless healing outcomes in humans.

Although not yet studied in amphibians, the type 1 cytokine hormone, leptin, is an important wound healing factor in mammals (12). Leptin signaling at the site of a cutaneous wound increases the local inflammatory response and cell recruitment (13). Later in the healing process, leptin increases re-epithelization through increased proliferation and migration of keratinocytes, and it also promotes blood vessel regrowth (14). There are several potential sources of leptin for these processes, including circulation and production at the wound site itself, which suggests that leptin may support wound healing in a variety of ways (15). In wild-type mice, leptin increases wound epithelialization and angiogenesis, while in *ob/ob* leptin-deficient mice, which have difficulty healing epidermal wounds, leptin treatment rescues epithelial proliferation and improves wound outcomes (16, 17).

Little is known of leptin’s actions in wound healing outside of mammals, but there is growing evidence that at least some of its immune roles are conserved (18). In lizards, leptin regulates energy allocation between reproduction and the immune system and rescues immune function in food-restricted lizards (19–21). In amphibians, leptin receptor (long form, LRb) mRNA is highly expressed in the juvenile skin (22), suggesting that leptin signaling could modulate actions of the skin and potentially be involved in wound healing. As in mammals, the amphibian leptin receptor signals through the stereotypical JAK/STAT pathways (23), which are activated during wound healing and pro-inflammatory responses in mammals. Finally, in the context of appendage regeneration, leptin mRNA is one of the most highly upregulated transcripts within 6 h of tail injury in the *Xenopus* tadpole (24, 25), as well as after optic nerve and spinal cord injury in newly metamorphosed froglets (26, 27).

In this study, we tested the hypothesis that leptin promotes cutaneous wound healing in juvenile *Xenopus laevis*, a model amphibian species. First, we examined the expression of leptin and leptin receptors in the dorsal skin. We then used cultured whole-skin explants to determine whether increasing paracrine leptin signaling enhanced wound healing and where leptin and activated leptin receptor were expressed after injury. To determine if circulating leptin levels and nutritive status prior to injury affect wound healing, we also pretreated fed and food-restricted frogs with leptin and assayed wound healing of skin explants in our culture system. We expected food restriction to impair wound healing and leptin pretreatment to rescue that effect.

## Methods

### Experimental animals

All experimental procedures were approved by the Institutional Animal Care and Use Committee of Washington State University. *X. laevis* frogs were obtained from Xenopus 1 (Dexter, MI, USA) as larvae and raised to 6–8 months post-metamorphosis. Animals of this age have recovered much of their immune function after the downregulation that occurs during metamorphosis but still retain full ability to regenerate skin scar-free after injury (9, 10). Frogs were fed every other day unless noted otherwise. In studies of *in vivo* wound closure in *Xenopus* juveniles, both incisional and square wounds form a simple, undifferentiated wound epidermis within 24–48 h of the wound occurring (9, 28). This is followed by epidermal thickening with mononuclear cells and cells from subdermal layers infiltrating to remodel the dermis (9, 28, 29). Scarless wound healing takes approximately 2 months in the juvenile, but remodeling takes much longer in the adult frog and will scar (9).

### Leptin and leptin receptor expression in *Xenopus* dorsal skin

To determine the expression profile of leptin protein in uninjured dorsal skin, we dissected skin from juveniles and fixed it in 3% paraformaldehyde (PFA)/1% glutaraldehyde overnight at 4°C; approximately 1 mm × 1 mm squares were cut from dorsal tissue and sectioned for immunohistochemistry (n = 6). To determine where the leptin receptor is expressed in the skin and to see if leptin activation of the receptor activates JAK/STAT signaling in the skin, 6–8-month *X. laevis* juveniles were injected with either sterile saline or 200 ng/g body weight recombinant *Xenopus* leptin protein (rXleptin) intraperitoneally (IP). Recombinant *Xenopus* leptin was produced following methods in Crespi and Denver (22) and purified using the Ni-NTA Purification System (K95001, Invitrogen, Waltham, MA, USA) in hybrid conditions following the manufacturer’s instructions. After 6 h, frogs were euthanized, and the dorsal skin was fixed and prepared for immunohistochemistry as described above (n = 3/injection type).

### *In vitro* wound healing assay culture conditions

To determine the effect of upregulation of leptin signaling on cutaneous wound healing, we developed an *in vitro* culture assay in which we incubated skin explants with or without recombinant *Xenopus* leptin. Six- to eight-month post-metamorphic *X. laevis* frogs were euthanized *via* benzocaine overdose followed by double pithing. Following methods adapted from Meier *et al.* (30), donut-shaped “punch-in-a-punch” *in vitro* wounds were created with cut tissue on the outside and inside edges. Briefly, dorsal skin was dissected from the neck and down the lateral lines to the lower back and then rinsed three times with sterile phosphate-buffered saline (PBS). The dorsal skin sheet was then placed on a sterile cutting mat, with care taken to minimize mucus production and damage to the hypodermis. After the skin was allowed to dry briefly and excess water to evaporate, a 4-mm tissue biopsy punch (World Precision Instruments, Sarasota, FL, USA) was used to remove circular sections of skin, which then had 1-mm biopsy punches removed from the center to create a wound and forming the skin donut. With care taken not to stretch the wound in the center, these tissues were placed in 24-well plates with either Matrigel (Corning Life Sciences, Glendale, AZ, USA) or ECM Gel (a Matrigel equivalent; E1270, Sigma-Aldrich, St. Louis, MO, USA), both of which are produced from Englebreth-Holm-Swarm murine sarcoma; 250 µl of each was diluted to 2.5 mg/ml protein with 1× L-15 media. After the skin donut explants were allowed to adhere to the basement layer for approximately 3 min, each well was flooded with 1 ml of sterile L-15 with 10% fetal bovine serum (FBS), 100 units/ml of penicillin, and 100 µg/ml of streptomycin. The tissue was incubated at 28°C. For immunohistochemistry, tissues were fixed at 0, 24, and 48 h post injury (hpi) with 3% PFA/1% glutaraldehyde overnight at 4°C.

### Leptin and pSTAT3 expression in wounded skin explants

To test if leptin protein and pSTAT3 activation patterns were changed at the site of injury, protein expression was visualized using immunohistochemistry. After fixation, the skin donut was cut through the center wound into pieces and assayed for leptin protein (n = 6) and pSTAT3 upregulation (n = 3), as well as cell proliferation (n = 3; see *Immunocytochemistry* methods below). Tissues were chosen from animals with no infected culture wells at 48 hpi to ensure an even sample size across all treatments and to minimize variation from individuals. Tissues were cryosectioned and evaluated for protein expression and cellular proliferation 1) within 300 μm of the edge of the inner wound of the donut and 2) the edge resulting from the cut made after fixation (post-fix) to determine if staining resulted from edge effects, and if leptin and pSTAT3 expression changed throughout the whole explant or just at the wounded edge.

### Leptin effects on skin explant wound healing assay: Leptin dose–response experiment

To test if an increase in leptin signaling affected the speed of wound closure, wounded dorsal skin donuts were incubated with 0, 10, or 100 ng/ml of rXleptin in both the Matrigel basement layer and media. Because we do not yet have a validated ELISA for *Xenopus* leptin, we do not know the circulating concentrations of leptin in frogs, so these doses were used based on prior experiments that showed leptin activation of cell proliferation in cultured *Xenopus* tadpole limbs and primary cultures of splenocytes (22, EJC, *unpubl. data*). Explants from every frog were included in each treatment, and tissues were assigned haphazardly to each treatment. Explants were imaged at 0, 24, and 48 hpi with a Leica S9i light microscope. Wells that became turbid with microbial infection were recorded, and only those explants from individuals with no infected wells were included in the wound closure assay and immunohistochemistry (initial individuals n = 24, wound closure assay analysis n = 12). The area of the wound opening was measured using ImageJ for each time point, and the percent closure was calculated based on wound size at 0 hpi; these images were analyzed blindly to treatment. All samples for this experiment were processed within a few days. Due to chance differences in skin adjustment in culture, the area of the circular wound at the start of the experiment varied but was not significantly different between treatments (0.307–0.737 mm^2^, one-way standard least squares ANOVA F_2,11_ = 1.3163, p = 0.3072); therefore, percent wound closure at 24 and 48 h was assessed with repeated-measures MANOVA with treatment, time point, and time * treatment as factors, followed by Tukey’s honestly significant difference (HSD) multiple-comparisons tests (α = 0.05).

### Effects of nutritional state and leptin administration on skin explant wound closure

To determine whether circulating as well as paracrine levels of leptin affect wound closure ability, we conducted an experiment in which we compared epidermal wound healing responses of daily fed and food-restricted frogs that were pretreated with either intraperitoneal injections of saline or leptin prior to euthanasia. Food restriction is commonly used to reduce endogenous leptin in circulation (31, 32), but this experiment also models either stress- or sickness-induced anorexia that commonly occurs in amphibians. We predicted that food restriction would slow wound healing and that endocrine leptin signaling would prime the epidermal explants for a stronger wound healing response. We also treated explants with leptin in the culture media to test whether local leptin signaling would increase wound healing rate in an additive fashion. Frogs were either fed every day or food restricted for 31 days, which is when circulating glucose and corticosterone levels are low and *Xenopus* juveniles begin to lose body fat (33); therefore, we expected that if a food-restricted state and presumably lower circulating leptin levels affect wound closure, it would occur after this time. After the 31 days, frogs in each group were given IP injections with either 30 µl of sterile saline or 200 ng/g frog weight rXleptin in 30 µl of sterile saline every other day for 7 days (four injections/animal; n = 8/fed status/injection type). All samples for this experiment were processed within a few days. The day following the last injection, animals were euthanized by benzocaine overdose, and their dorsal skin was removed for wound healing assays as described above. Dorsal skin donuts were incubated with or without rXleptin (100 ng/ml) in ECM Gel in L-15 media for 48 hpi; imaged at 0, 24, and 48 hpi; and fixed in 3% PFA/1% glutaraldehyde overnight at 4°C for immunohistochemistry. Only animals that had explants in all treatments and did not have visible microbial infection were included in the analysis (n = 8/treatment group). We also saw variation in the initial area of wounds similar to the dose–response experiment with no significant difference between treatments (0.302–0.722 mm^2^, one-way standard least squares ANOVA, F_3,50_ = 1.7113, p = 0.1766). In this experiment, we compared the slope of wound sizes from 0 to 48 hpi for each individual; measurements made were not blind to treatment groups. We then used three-way standard least squares ANOVA to determine the effects of nutritional status, pretreatment, culture condition, and their interactions on wound closure; if two- and three-way interactions were not significant, we removed them from the model. Significant differences among treatment groups within each time period were then evaluated using Tukey’s HSD multiple-comparisons tests (α = 0.05).

### Effect of leptin on microbial growth in skin explant culture

During the wound healing experiments, we observed that explants cultured without leptin became microbial infected more frequently as compared to those cultured with leptin (note that explants that were infected were not included in the above analyses). As a *post-hoc* analysis, we compared infection frequency (i.e., whether culture media was visually cloudy) at 48 h of culturing between saline and rXleptin using a likelihood ratio test. Because L-15 media changes color with pH, we could not use absorbance assays to quantify the extent of infection. We included all explants cultured across experiments except for food-restricted animals because there was only one infected well in this set of explants. Sample sizes per dose were as follows: 0 ng/ml, ln = 70; 10 ng/ml, n = 38; 100 ng/ml, n = 70.

### *Xenopus* leptin antibody and pre-absorption

To detect leptin protein, we produced a *Xenopus*-specific leptin polyclonal antibody raised in rabbits injected with purified recombinant *Xenopus* leptin protein in the Washington State University Center for Reproductive Biology Core Facility. To ensure that this anti-leptin polyclonal antibody (anti-Xleptin) was binding to leptin specifically, the anti-Xleptin was incubated 1:10 with 400 ng/µl of rXleptin protein at room temperature for 1 h prior to primary staining as described below (pre-absorption).

### Immunohistochemistry

Immunohistochemistry was used to describe the cellular localization of leptin protein and pSTAT3 as an indicator of leptin receptor activation within the skin (injured and not injured) and phospho-histone H3 (PH3) to detect proliferating cells. All fixed tissues were dehydrated with methanol for storage at −20°C and then rehydrated stepwise in PBS with 0.01% Triton X-100 (PBST). They were bleached in a solution consisting of 5% hydrogen peroxide (30% stock) and 5% formamide in PBS on a light table for 2 h until granular glands were pale cream in color and all other tissues were white. Tissues were rinsed 3 × 2 min in PBST followed by 2 × 30 min permeabilization in PBST. Tissues were blocked in 10% blocking reagent (Roche 11096176001, Sigma-Aldrich, St. Louis, MO, USA) in PBST for 1 h at room temperature and then incubated overnight at 4°C with the primary antibody in 10% blocking reagent in PBST at the following concentrations: 1:2,000 anti-Xleptin, 1:250 anti-phosphohistone H3 (PH3, ab14955, Abcam, Cambridge, UK), 1:500 phospo-STAT3 (pSTAT3; 9145, Cell Signaling Technology, Danvers, MA, USA), or 1:500 acetylated alpha-tubulin (AαT; T7451, Sigma-Aldrich). Tissues were washed 3 × 10 min in PBST, then re-blocked for 1 h as above, and incubated with 1:500 secondary antibody in 10% blocking reagent in PBST for 2 h at room temperature (Alexa Fluor goat anti-rabbit 647, goat anti-mouse 647, or goat anti-mouse 488, Invitrogen, Waltham, MA, USA). Finally, tissues were rinsed 3 × 10 min in PBST. If tissues were for cross sections, they were perfused with 15% sucrose/15% cold water fish gelatin and rocked overnight at room temperature, mounted in Tissue-Tek O.C.T. compound (4583, Secura Finktek USA, Torrance, CA, USA), and sectioned at 16 µm. Following sectioning, tissues were washed with PBS and mounted using VectaShield Antifade Mounting Media with DAPI (H-1200-10, Vector Laboratories, Burlingame, CA, USA). Whole-mount tissues were dehydrated in 100% methanol and cleared using 1:2 benzyl alcohol:benzyl benzoate. Both sections and whole-mount tissue were imaged using a Leica SP8 confocal microscope.

### Statistical Analysis

All statistical tests and graphical visualization of data were performed using JMPPro v.16. Data were assessed for normality prior to any other analysis.

## Results

### Leptin and pSTAT3 expression in dorsal skin

**Figure 1:**
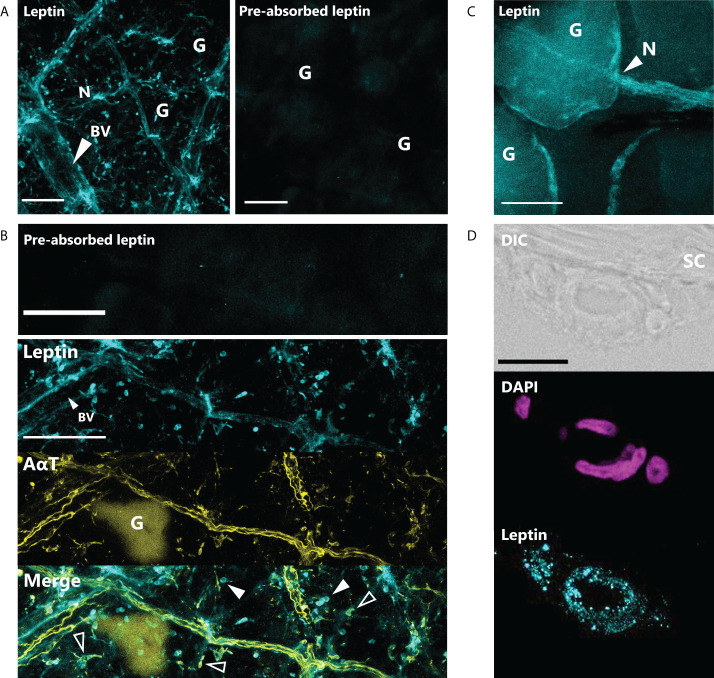
Leptin is expressed in nerves and blood vessels in the dorsal skin of *Xenopus laevis* juveniles. **(A)** Leptin protein is expressed in blood vessels (BV) and nerves (N) in the dermis and hypodermis. Only background fluorescence from granular glands (G) is present in tissue stained with antibody that was incubated with recombinant *Xenopus* leptin protein (4 μg) prior to staining (pre-absorbed). Scale bars, 100 µm. **(B)** Leptin is expressed in the endothelial cells of a large blood vessel (BV) in the hypodermis and unknown scattered cell bodies (solid arrows). Leptin also co-localizes with acetylated alpha-tubulin (AαT, yellow), a neural marker in axons and some nerve cell bodies (hollow arrows). Granular glands are autofluorescent after bleaching and clearing at 488 nm (as seen in the AαT panel). Scale bars, 100 µm. **(C)** Leptin protein co-localizes with axons innervating a granular gland. **(D)** Cross section showing leptin in the endothelial cells of a blood vessel in the hypodermis, up against the stratum corneum (SC) of the dermis. Scale bar, 25 µm.

**Figure 2:**
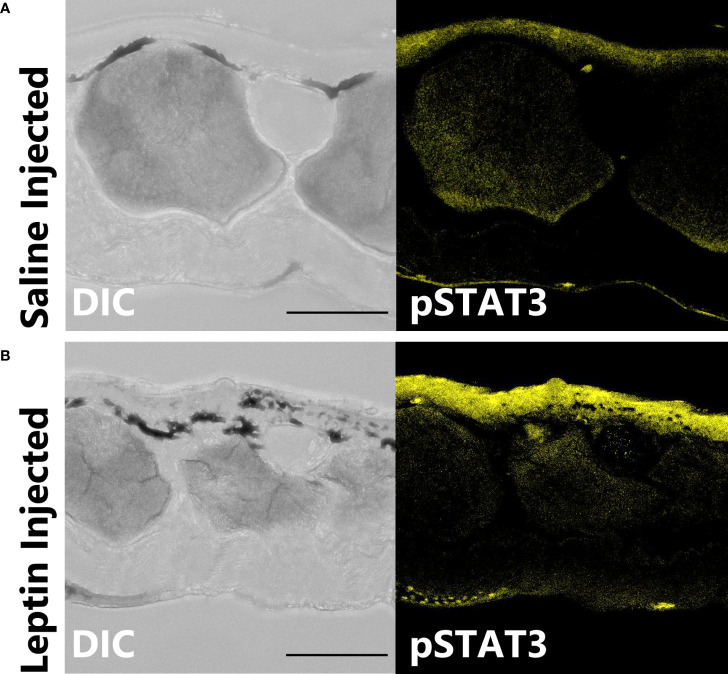
Leptin stimulates JAK/STAT signaling in skin. Leptin-induced phosphorylated STAT3-ir (pSTAT3-ir, yellow) in 16-µm sections of juvenile *Xenopus laevis* dorsal skin; skin was fixed 6 h after intraperitoneal injection with **(A)** saline or **(B)** leptin. Leptin stimulates pSTAT3-ir strongly in the epidermis and at the apical end of the granular glands and hypodermis (representative image from n = 3). Scale bar, 100 µm **(**n = 3). Epidermis (E), dermis (D), stratum corneum of the dermis (SC), and thin hypodermis (H). Scale bar, 50 µm.

Leptin protein was found to be widely expressed in the nerves and blood vessel network of the dermis and hypodermis, as well as other unidentified cell types in the hypodermis of juvenile *X. laevis* dorsal skin (**Figure 1**). Large, major blood vessels in the dermis had nerves running closely alongside (shown by AαT-ir), and leptin-ir co-localized with these nerves (**Figures 1A, B**). Leptin protein was found in axons that innervated granular glands (**Figure 1C**) and specifically in the endothelial tissues of blood vessels (**Figure 1D**). Leptin-ir staining was not apparent in the rXleptin-pre-absorbed samples (**Figures 1A, B**), supporting that this expression pattern is specific to leptin protein. We also detected leptin-induced pSTAT3-ir in the epidermis and hypodermis, with some staining on granular glands, indicating that leptin receptor is present in these cells adjacent to those in which leptin protein is produced, and JAK/STAT signaling is activated in these tissues (**Figure 2**).

### Leptin and pSTAT3 expression in wounded skin explants

**Figure 3:**
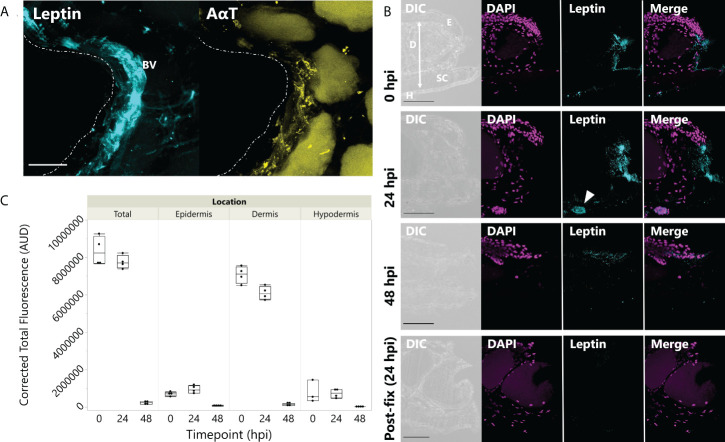
Leptin protein is rapidly localized to injured skin. **(A)** Whole mount of leptin protein (aqua) and acetylated α-tubulin (AαT, yellow) at the time of injury in juvenile *Xenopus laevis* dorsal skin. Dotted line indicates injury at the center of the donut-shaped explant. Leptin is highly expressed in blood vessels and nerve tissue (indicated by AαT) associated with the injury site. Granular glands are autofluorescent after bleaching and clearing at 488 nm (as seen in the AαT panel). Scale bar, 100 µm. **(B)** Leptin protein expression in 16-µm cross sections of juvenile *X. laevis* dorsal skin after injury. Leptin expression (aqua) is concentrated in the injured tissue at 0 and 24 hpi and nearly depleted by 48 hpi *in vitro* except for some localized in the epidermis (DAPI, magenta). Epidermis (E), dermis (D), stratum corneum of the dermis (SC), and thin hypodermis (H). Arrow in 24 h panel indicates blood vessel. Scale bars, 100 µm. **(C)** Leptin is most highly expressed in the central dermis after injury, but expression within 300 µm of the injury site in all skin layers is depleted by 48 hpi in culture (standard least squares ANOVA, time point, p < 0.0001).

**Figure 4:**
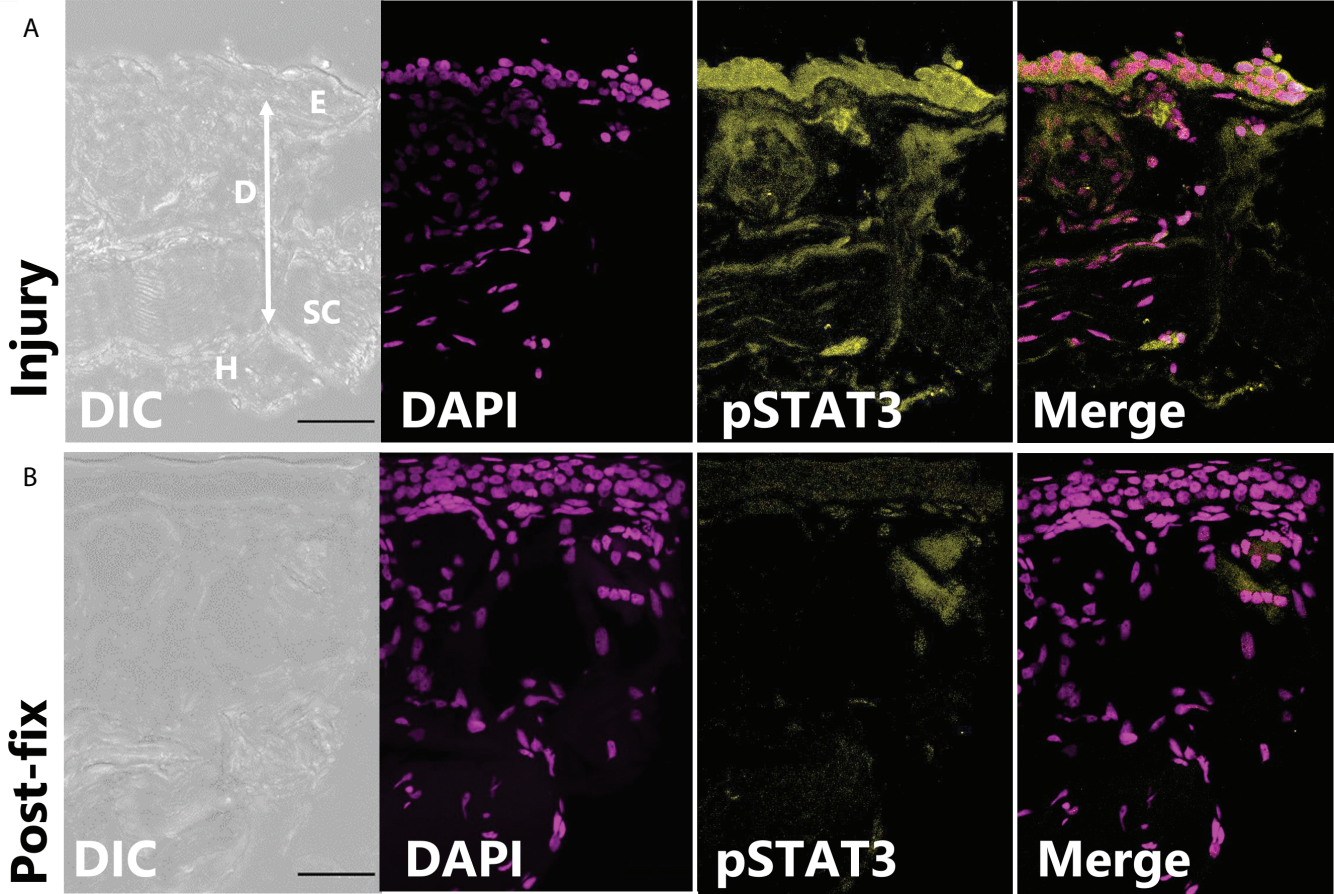
pSTAT3-ir increases after full thickness injury of dorsal skin. **(A)** The highest pSTAT3 activation was associated directly with the injured edge of the explant (integrated density: 589,578 ± 201,764 ADU, n = 3). **(B)** By contrast, the end of tissue explant that was non-injured and cut after fixation had less pSTAT3-ir activation (integrated density: 267,042 ± 45,092 ADU).

In tissues fixed immediately after injury, leptin protein-ir was already found concentrated at the wound site, particularly associated with bisected blood vessels and nerves (**Figure 3A**). Leptin expression at the injury site, which appeared primarily extracellular, was significantly higher between 0 and 24 hpi, with nearly all leptin being depleted at 48 hpi except for some leptin-ir in the epidermal layer of cells (**Figures 3B, C**; standard least squares ANOVA, skin layer, F_5,41_ = 26.7972, p < 0.001). In tissue cross sections from the same explants, post-fix cuts did not have strong leptin expression in the tissue or at its edge, indicating that leptin protein was specifically concentrated at the site of injury and was not an “edge effect’ of antibody penetration (**Figure 3B**). This leptin-ir co-localized with pSTAT3-ir expression in the wounded explant, which was highest in the epidermis (like the uninjured skin), but also expressed in the granular gland, dermis, and hypodermis at the wound site compared to a post-fix cut in the middle of the explant (**Figure 4**). We did not detect PH3-ir cells in our sections, suggesting that cells were not yet undergoing mitosis at 48 hpi (data not shown).

### Leptin effects on skin explant wound closure

**Figure 5:**
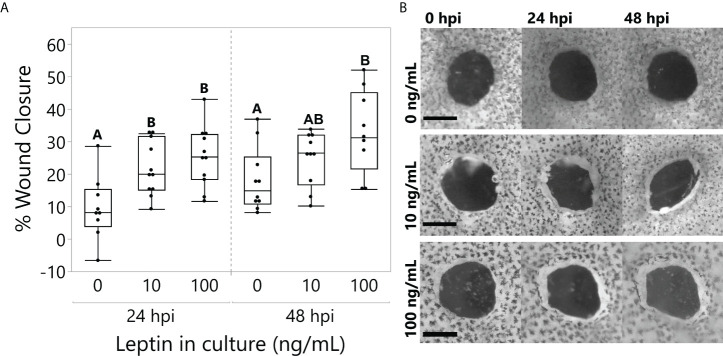
Leptin treatment (0, 10, and 100 ng/ml) in culture media significantly improved wound healing in juvenile *Xenopus laevis* dorsal skin explants at 48 h of culture. **(A)** At 24 h, treatments at both 10 and 100 ng/ml significantly increased wound closure compared to the control, and by 48 h, the 100 ng/ml treatment continued to improve wound healing compared to the control (repeated-measures MANOVA, treatment = 0.0034, Tukey’s HSD, p < 0.05, n = 12/treatment). **(B)** Representative samples of dorsal skin explants in culture showing the interior wound of the “punch-in-a-punch” donut-shaped biopsy. Wound closure measured as the total area reduction of the central wound compared to 0 hpi. Scale bars, 0.5 mm.

All explants partially closed over time (time, F_1,25_ = 13.4764, p = 0.0011), but those cultured with 10 and 100 ng/ml of leptin closed to a greater extent at 24 hpi; only 100 ng/ml closed significantly more than the control at 48 hpi (**Figure 5**; ANOVA Treatment F_2,25_ = 7.2136, p = 0.0034).

### Effects of nutritional state and leptin administration on skin explant wound closure

**Figure 6:**
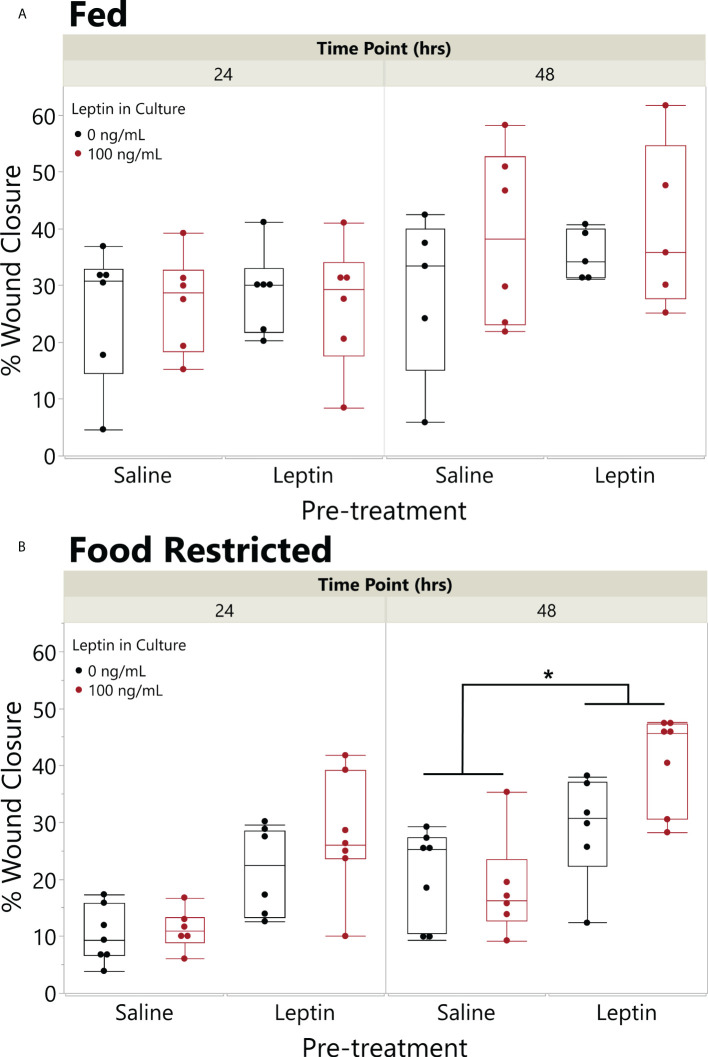
Pretreatment with leptin rescues effect of food restriction on wound closure *in vitro*. Effect of leptin on *in vitro* wound healing of juvenile *Xenopus laevis* dorsal skin explants where animals were fed **(A)** once a day or **(B)** food restricted for 31 days and then injected in the peritoneum (IP) with leptin (200 ng/g body weight) or saline every other day for 7 days. Dorsal skin donut explant wounds were then treated with (black) or without leptin (red) in culture (100 ng/ml) for 48 h. Pretreatment with IP leptin injection significantly increased the rate of wound closure in food-restricted animals over those with pretreatment injections of saline by 48 h, but there was no effect of pretreatment injection in fed animals (ANOVA, fed status * injection, p = 0.0253); asterisk indicates significant increase in wound healing in leptin pretreated vs. saline pretreated explants (Tukey’s HSD, p < 0.05, n = 8/treatment). There was no significant effect of leptin in the culture media (black vs. red) in this model (p = 0.1809).

On average, extended food restriction resulted in 18% weight loss (wet weight mean ± SD, start 11.20 ± 3.61 g, end 9.20 ± 2.69 g), while the fed daily group maintained its body weight (8.61 ± 3.66 g, end 8.67 ± 3.32 g). The effect of food restriction on wound closure depended on leptin pretreatment (ANOVA fed status * pretreatment, F_4,41_ = 5.3898, p = 0.0253). In explants from frogs fed daily, there was no effect of leptin pretreatment or leptin in the culture media on wound closure rate, although explants treated with leptin in culture exhibited the fastest closure rates by 48 hpi (**Figure 6A**). By contrast, in explants from food-restricted frogs, leptin pretreatment significantly increased wound closure rates as compared to those injected with saline (**Figure 6B**). Although leptin addition to the culture media tended to further increase wound closure rates, we did not detect an overall effect of *in vitro* leptin treatment (**Figure 6B**).

### Effect of leptin on microbial growth of skin explant culture

**Figure 7:**
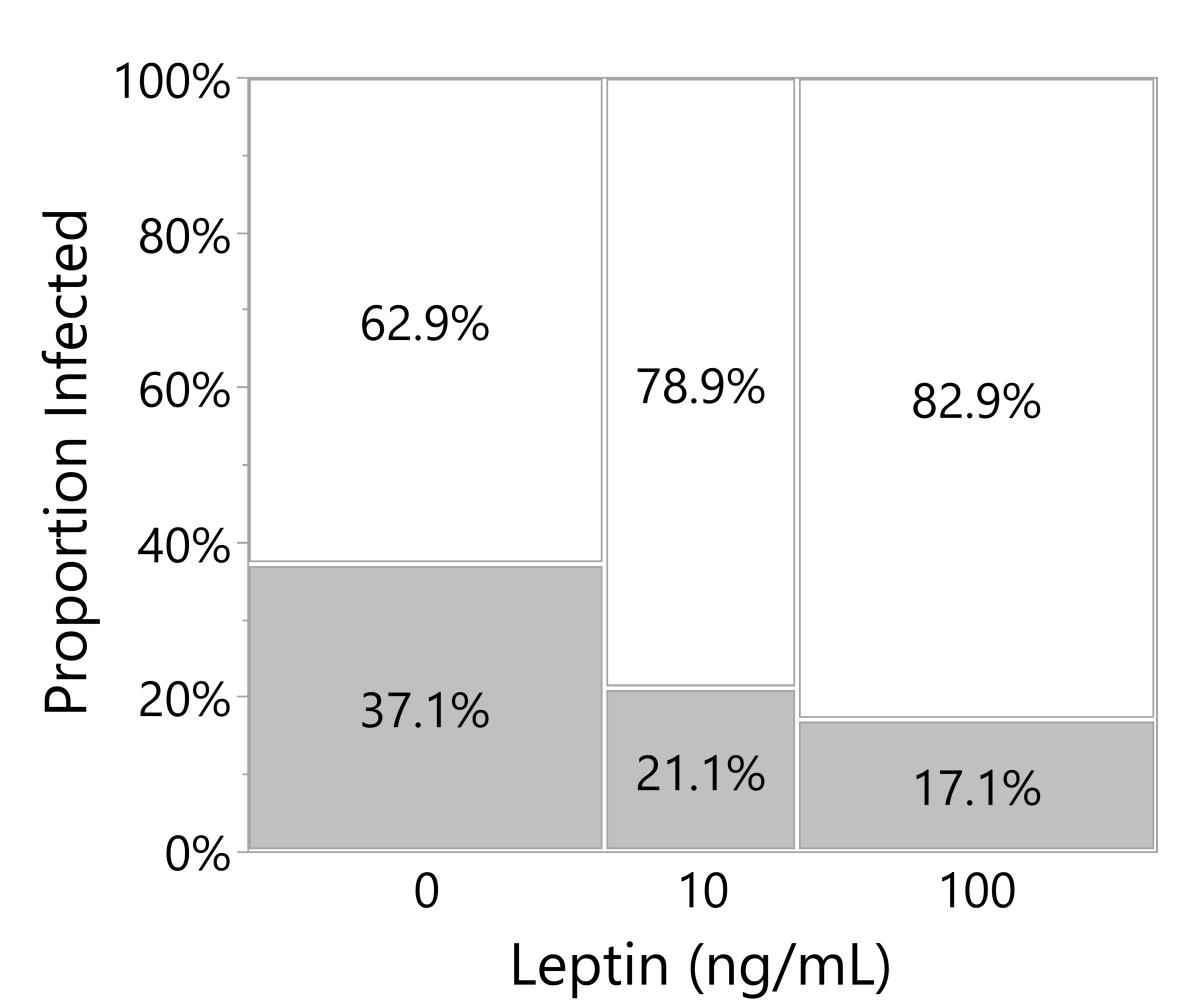
Leptin suppresses microbial growth in culture media of skin explants. Proportion of culture wells visually turbid with bacterial infection for juvenile *Xenopus laevis* dorsal skin wound explant cultured in increasing leptin concentration at 48 hpi. Increasing leptin concentration (0 ng/ml, n = 70; 10 ng/ml, n = 38; 100 ng/ml, n = 70) significantly decreased the number of infected wells (likelihood ratio test, p = 0.0202).

Across both experiments analyzed in this *post-hoc* analysis, leptin treatment in the culture media decreased the number of culture wells with microbial growth (likelihood ratio test, p = 0.0202; note that infected wells were excluded from the above analyses). Leptin at 100 ng/ml reduced the proportion of wells infected by almost half when compared to wells with no exogenous leptin added (**Figure 7**).

## Discussion

In this study, we show that leptin protein is expressed in the skin, released after injury at the site of a wound, and improves wound closure in *X. laevis* juvenile frogs. In uninjured skin, leptin protein was present mainly in the nerves and endothelial cells of blood vessels in the hypodermis prior to the injury, yet leptin activates JAK/STAT signaling (leptin-induced pSTAT3 upregulation) most strongly in the epidermis, but also the dermis, the hypodermis, and the apical surface of the granular glands. After injury, we localized leptin and pSTAT3 in all three skin layers within minutes after injury with the highest concentration in the epidermis and dermis at the edge of the injury. Lastly, we showed that the rate of wound healing in our *in vitro* assays increases with elevated leptin signaling; and when wound healing is slowed in explants harvested from food-restricted frogs, systemic leptin administration rescues wound closure ability to rates similar to those of fed frogs. These findings complement studies showing upregulation of leptin mRNA at the site of wounds in larvae (24–27) and provide the first functional evidence that leptin signaling modulates wound healing in amphibians.

When combined with findings showing that topical leptin enhances wound healing in mammals and lizards (14, 16, 34), our findings are consistent with the idea that leptin signaling is an ancient, evolutionarily conserved modulator of wound healing in vertebrates through both endocrine and paracrine signaling. In both *ob/ob* and food-restricted mice, wound closure is delayed compared to wild-type mice, but topically applied leptin increases STAT3 phosphorylation and reverses this outcome (16, 35). In our study, we show that leptin is present in neurons and endothelial cells of the blood vessels in the skin, which allow immediate leptin release at the site of the wound, although we cannot rule out delivery of leptin through the blood to the site of the wound. We also observed that food restriction slowed wound healing relative to well-fed animals, as shown in mammals (36). The lack of effect of leptin in fed-daily frogs could be due to elevated endogenous nutritional modulators or that leptin receptor levels were saturated such that additional leptin signaling has no effect. Conversely, extended food restriction could have depleted endogenous wound healing factors that minimized the effect of topical leptin treatment as shown in other experiments (e.g., 22), such that topical leptin treatment could not potentiate wound healing as shown in the dose–response experiment. Experimentally elevating circulating leptin levels reversed the effect of food restriction on wound healing, suggesting that endocrine leptin signaling prepares the skin for robust wound healing responses. This pretreatment appeared to reduce the effect of topical leptin in culture or reduced its effect size (there was only a trend of an additive effect of both leptin in food-restricted frogs, perhaps due to the same factors limiting leptin effects on wound healing in fed frogs). At this time, however, we were unable to measure leptin in circulation due to the lack of a functional ELISA at this time, but future research will allow us to further explore paracrine vs. endocrine contributions of leptin signaling in the context of nutritional regulation of wound healing in amphibians.

Nevertheless, the cellular mechanisms through which leptin signaling promotes wound closure remain unclear. While we observed variation in the amount of epidermal formation along the wound edge, our choice to support the explant culture with Matrigel or ECM matrix and minimal culture media made it not possible to image epithelial tongue formation, and the lack of PH3 staining may indicate that leptin alone cannot support proliferation of epidermal cells within 48 h of wounding. Similarly, in human corneal epithelium monolayers, it has been shown that leptin does not stimulate proliferation in growth media-starved cells (37). In a prior study, adult *Xenopus tropicalis* skin explants closed more quickly and formed epithelial tongues when supported with 5% frog serum supplemented with insulin compared to controls at both 24 and 72 h (30). Thus, leptin alone may not be enough to stimulate cell migration or proliferation and must be supplemented with insulin or other growth factors. However, our finding of leptin-induced JAK/STAT signaling in the epidermis, which is also elevated after injury, is consistent with leptin’s role in keratinocyte proliferation during wound healing in mammals (16, 17).

If the closure we observed was not the result of cell proliferation, there are several alternative mechanisms that can be tested. First, leptin has been shown to increase cellular migration in both cancer and wound healing contexts in mammals (14, 38), and future studies could involve tracers to better track cellular movements. Second, leptin capacitates pro-inflammatory and innate immune responses in the epidermis *via* JAK/STAT signaling pathways, which are highly upregulated by leptin signaling in our study. Leptin is a growth factor for monocytes, boosts the release of TNF-α and IL-6 from resident macrophages, and stimulates dendritic cell responses (39, 40). Our *post-hoc* observation that leptin reduced the frequency of microbial infections in our explant assays may be related to these actions. Future experiments could be designed to test the hypothesis that leptin is increasing the pro-inflammatory immune response to resist infection, reduce necrosis, and prevent uncontrolled bacterial growth in the skin. A final hypothesis for why the tissue explants treated with leptin exhibited high amounts of wound closure in such a short period is that leptin may have a role in wound contractility. This phenomenon has not been well studied in amphibians, but it has been shown in the North American bullfrog (*Rana catesbeiana*) that the ability of wounds to close by contraction peaks in late-stage prometamorphic tadpoles and is much less in adults, though no information on the juvenile stages is available (41). Studies in mice have shown that wound contraction is significantly decreased by anti-leptin application on epidermal wounds (42) and it is also reduced in *ob/ob* mice, which has been hypothesized to be linked to decreased collagen (43). Further studies are necessary to understand the mechanisms that link leptin to wound contraction.

Our study is the first description of leptin protein expression in the hypodermis, a thin layer of connective tissue, blood vessels, and nerves below the dermis in post-metamorphic frogs. The co-localization of leptin protein-ir and the neural marker AαT-ir shows that leptin is in the nerves throughout the skin, some of which connect to granular glands. This suggests that neurosecretory leptin could have a role in mediating antimicrobial peptide (AMP) production or secretion. AMPs are one of the most important immune defenses against skin infection, and given that microbial growth was reduced in frequency in leptin-treated explants, it is possible that there was an increased release of AMPs in our leptin-treated explants. Leptin has been shown to mediate secretion, particularly mucous production/secretion, in other mammalian contexts (44, 45), so it is possible that leptin enhanced wound closure in our experiments through these cytoprotective and antimicrobial actions.

We also showed leptin-ir in blood vessels and in cells that have not been described in the hypodermis layer. The hypodermis of mammalian skin contains a layer of subcutaneous fat that secrete factors, such as leptin, to modulate the function of the dermis and epidermis, which is the major source of leptin in mammals, but amphibians lack an organized subcutaneous fat layer (46). While we did not conduct histology in the skin from fed daily or food-deprived frogs, we plan on repeating that study to determine if these leptin-positive cells are indeed scattered fat cells, which could be another source of leptin that varies with nutritive conditions in frogs. This study highlights the potential modulatory roles of the hypodermis in wound healing in frogs, a layer that is largely ignored in the literature (46).

Because we focused on wounded skin explants in this study as the first test of leptin's actions during wound healing, there were some limitations to our ability to fully explore the roles of leptin signaling. For example, it is possible that leptin treatment alone was not enough to stimulate mitosis in our culture conditions, or that leptin acts as a mitogen during later stages of wound healing, after the 48 hpi period. Leptin depletion in the tissue by 48 hpi *in vitro* limited the duration of this study to explore actions that extend in later stages of wound healing. In mammals, leptin mRNA has been shown to be upregulated for several days after a wound (15) when it continues to promote keratinocyte proliferation and angiogenesis (14, 16, 35). Leptin protein expression in endothelial cells of blood vessels in the hypodermis suggests that leptin plays a role in angiogenic regrowth into a wound. Furthermore, endocrine sources of leptin could sustain leptin-mediated processes involved with these later stages of wound healing. This study confirms that leptin is present and is likely a paracrine, neurosecretory, or endocrine signal that promotes wound closure in amphibians, justifying that further work is needed to elucidate through which mechanisms it is acting and to confirm these effects *in vivo*.

These findings have important implications for the veterinary care of captive amphibians. Hundreds of amphibian species are kept as pets, in zoos, including assurance colonies of endangered or threatened species of amphibians (47), and in the research laboratory (e.g., *Xenopus*). During transport or captivity, amphibians are vulnerable to injuries, and their wounds are often fatal due to secondary infections from ubiquitous gram-negative bacteria, such as *Aeromonas*, combined with sickness-induced anorexia (1–3). Furthermore, fungal pathogens such as *Batrachochytrium dendrobatidis* or *Batrachochytrium salamandrivorans* invade the skin, and prolonged infections are highly lethal in many species of frogs and salamanders (6, 48–50). Factors that improve wound healing and immunity broadly across amphibians are needed (3), especially those that do not cause adverse side effects or concerns about persistence in wastewaters. This study provides evidence that supports leptin as an emerging candidate for novel prophylactic or post-injury therapies that veterinarians, zoo curators, and caregivers can use to accelerate wound healing to improve the health and survival of amphibians. The fact that IP injection of leptin, which can be done quickly with minimal handling and discomfort, increases wound healing as much or more than topical application widens the application of this to fully aquatic species, especially aquatic species where topical applications could be diluted. Because these wound healing effects of leptin appear to be evolutionarily conserved, leptin-based approaches could be applied to diverse groups of vertebrates.

## Data availability statement

The raw data supporting the conclusions of this article will be made available by the authors, without undue reservation.

## Ethics statement

The animal study was reviewed and approved by Washington State University Institutional Animal Care and Use Committee.

## Author contributions

RR and EC contributed to the conception and design of the study, statistical analysis, and primary contributors to the draft of the manuscript. RR, KQ, and GC conducted laboratory research and confocal microscopy. All authors contributed to writing the manuscript and read and approved the final submission.

## Funding

This work was funded by the American Microscopical Society Summer Fellowship to RR, Washington State University College of Arts and Sciences Mini-Grant and Auvil Fellowship to KQ, Sigma Xi Grant-in-Aid of Research to GC, and NSF-DEB 1754474 to EC.

## Acknowledgments

The authors would like to thank JLB for assistance with statistical analysis, and DM, VLH, and the Franceschi Microscopy and Imaging Center at WSU for assistance with confocal microscopy.

## Conflict of interest

The authors declare that the research was conducted in the absence of any commercial or financial relationships that could be construed as a potential conflict of interest.

## Publisher’s note

All claims expressed in this article are solely those of the authors and do not necessarily represent those of their affiliated organizations, or those of the publisher, the editors and the reviewers. Any product that may be evaluated in this article, or claim that may be made by its manufacturer, is not guaranteed or endorsed by the publisher.
